
JOURNAL INFORMATION
==============================
NLM Title Abbreviation: ScientificWorldJournal
Journal ID: ScientificWorldJournal
NLM Journal ID: 101131163
Journal ID: TSWJ

The Scientific World Journal

ISSN: 2356-6140
EISSN: 1537-744X
Publisher: Wiley

ARTICLE INFORMATION
==============================
PMCID: PMC6009404
PMID: 29973814
DOI: 10.1100/tsw.2002.40
Article ID: 725093
Article version: 1
Subjects: Short Report

Identification of Protein Folding Cores Using Charge Center Model of Protein Structure

Torshin Ivan Y. *
Harrison Robert W.
Weber Irene T.
Petock John M.
Department of Biology, Georgia State University, Atlanta, GA, USA
*Ivan Y. Torshin: biotiy@suez.cs.gsu.edutiy1@mail.com
Publication date: 2002
Electronic publication date: 2002 Feb 5
Volume: 2
First page: 84
Last page: 86
Copyright: Copyright © 2002 Ivan Y. Torshin et al.
Copyright year: 2002
License: This is an open access article distributed under the Creative Commons Attribution License, which permits unrestricted use, distribution, and reproduction in any medium, provided the original work is properly cited.
License URL: https://creativecommons.org/licenses/by/3.0/

==============================
Advances in Gene Technology: The Genome and Beyond –
Structural Biology for Medicine (Proceedings of the 2002 Miami
*Nature Biotechnology* Winter Symposium)
*TheScientificWorld* 2002, 2(S2), 84–86
ISSN 1532-2246; DOI 10.1100/TSW.2002.40

### IDENTIFICATION OF PROTEIN FOLDING CORES USING CHARGE CENTER MODEL OF PROTEIN STRUCTURE

Ivan Y. Torshin*, Robert W. Harrison, Irene T. Weber, and John M. Petock
Department of Biology, Georgia State University, Atlanta, GA
*(biotiy@suez.cs.gsu.edu, tiy1@mail.com)

**INTRODUCTION**. Genome annotation (or “interpretation”) is the next important step in the so-called “postgenomic era”. Structural genomics enterprises (which provide high-throughput structure determination) and protein structure prediction are important methods for making the step. Both require specific “high-throughput” computational techniques that would allow efficient processing of large quantities of information. Further, in order to decipher already available spatial structure of a protein it is necessary to at least identify residues that support biological function(s) of the protein (i.e., active site) and the residues important for the folding.

**METHODS**. This presentation introduces a novel method for identification of the folding cores in proteins. Positive and negative charge centers, defined solely from the structure of the folded protein are likely to indicate the protein’s folding core. The residues and sequences, identified by the procedure, are highly consistent with the available biochemical data on folding as seen for a set of about 20 proteins[1]. Here, the folding of chymotrypsin inhibitor-2 (CI2) from barley is analyzed in some detail.

**RESULTS.** The charge center analysis[1] suggests four general stages of protein folding (illustrated in Fig. 1). Below, comparison with the biochemical data for CI2 is presented. Residues identified by the analytical procedure and those from the biochemical experiments are summarized in Table 1.

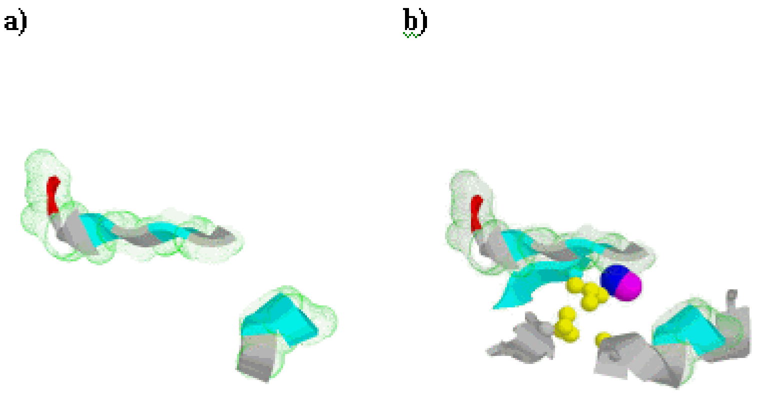

c) d)

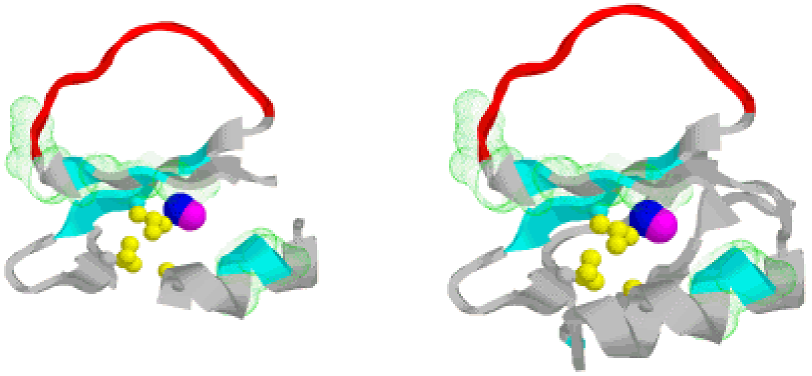

**FIGURE 1.** Stages of protein folding using CI2 as an example. a) *local hydrophobic collapse;* b) formation of the residue cluster(s) around the charge centers, then *electrostatic collapse;* c) *global hydrophobic collapse*, formation of the active/binding site; (d) *slow annealing* to the native state. HX (hydrogen exchange) residues (cyan), continuous hydrophobic sequences (green dots), residues of folding nucleus (yellow), charge centers (positive, blue sphere, negative, red) and the binding site (loop colored in red) are shown. See text for the details on biochemistry of CI2 folding.

**TABLE 1**
Residues around the positive and negative charge centers (CC+,CC-), of the hydrogen exchange core (HX) and of the folding nucleus (NMR) for CI2 from barley (PDB 1ciq). Only hydrophobic residues are listed. Conserved residues are marked with ‘*’. Residues in this table comprise less than 1/3 of all hydrophobic residues in CI2.

| CC+ | CC- | HX [2] | NMR[3] |
|---|---|---|---|
| W5* | W5 | | |
| L8* | L8 | | |
| A16* | A16 | | **A16** |
| I20* | I20 | **I20** | |
| | L21_ | **L21** | |
| I29_ | I29 | | |
| I30_ | I30 | **I30** | |
| | | **L32_** | |
| V47* | V47 | **V47** | |
| L49* | L49 | **L49** | **L49** |
| F50* | F50 | **F50** | |
| I57* | I57 | | **I57** |
| P61* | P61 | | |

Let’s consider folding of CI2 stage by stage. **1.** The analysis[1] suggests that *continuous hydrophobic sequences* (in CI2 there are 3 of them: 19-21 VIL, 29-35 IIVLPVG, and LFV 49-51) are likely to be the earliest folding events. Indeed, NMR experiments and molecular dynamics of CI2 unfolding[3] suggest “hydrophobic clustering” of the sequence 29-35 as well as 19-21 (which is part of the only helix, Fig. 1a). **2.** *Nucleation* site for folding of the CI2 was suggested to include regions of the protein around the residues Ala-16, Leu-49 and Ile-57[3]. These residues comprise the clusters around the charge centers (Table 1). Major event in the folding seems to be closing of the helix to the beta-sheet[3], which agrees with the charge centers being located between the helix and the beta-sheet (Fig. 1b) **3.** Hydrogen exchange cores are likely to be an important intermediate in folding[2]. *Global hydrophobic collapse* supposedly led to formation of the active/binding site[1], which at least correlates with the structure CI2 (Fig. 1c**,** the binding loop 54-64[4] [35-35 in 1ciq] is between the strands which are brought together in this stage). **4.** *Slow annealing* (Fig. 1d) is, perhaps, the longest stage in folding, and it would include minor repacking of the half-folded protein.

**DISCUSSION.** Although the role of electrostatics in folding has not been studied in detail[1], electrostatic interactions may be particularly important for the folding of CI2[5]. For 16 out of 20 proteins, at least 80% of the core sequences could be correctly identified using the charge center model[1]. It should be noted, that this result is based on an “ab initio”-type of calculation, which suggest solid physical chemistry behind the model. Basic physico-chemical considerations do suggest that electrostatic interactions are more effective in driving together distant sequence regions than a “blind search” of the most efficient packing of the hydrophobic core[1].

**CONCLUSION.** The analytical method, based on charge centers model, provides a detailed picture of the folding states. This picture is in good agreement with the available experimental data and it also provides additional details, which are not always available from experimental studies. Due to extreme simplicity of the calculation, the procedure is highly efficient computationally. Therefore, a method based on the charge center model could be used for identification of potential folding cores in large sets of protein structures.

**REFERENCES**

1. Torshin, I.Y. and Harrison, R.W. (2001) Proteins 43, 353–364.
2. Li, R. and Woodward, C. (1999) Protein Sci. 8, 1571–1590.
3. Kazmirski, S.L.,Wong, K.B., Freund, S.M.V., Tan, Y.J., Fersht, A.R., and Daggett, V. (2001) Proc. Natl. Acad. Sci. U. S. A. 98, 4349–4354.
4. Shaw, G.L., Davis, B., Keeler, J., and Fersht, A.R. (1995) Biochemistry 34, 2225–2233.
5. Tan, Y.J., Oliveberg, M., Davis, B., and Fersht, A.R. (1995) J. Mol. Biol. 254, 980–992.
